# Phenotypical Identification and Toxinotyping of *Clostridium perfringens* Isolates from Healthy and Enteric Disease-Affected Chickens

**DOI:** 10.1155/2023/2584171

**Published:** 2023-02-08

**Authors:** Eaftekhar Ahmed Rana, Tanvir Ahmad Nizami, Md Sayedul Islam, Himel Barua, Md Zohorul Islam

**Affiliations:** ^1^Chattogram Veterinary and Animal Sciences University, Khulshi, Chattogram-4225, Bangladesh; ^2^Bangabandhu Sheikh Mujibur Rahman Agricultural University, Gazipur-1706, Bangladesh

## Abstract

*Clostridium perfringens* is a ubiquitous spore-forming anaerobic pathogen that is frequently associated with enteric disease in chickens. Moreover, enterotoxin-producing *C. perfringens* has high zoonotic potential as well as serious public health concerns due to the emanation of food-borne intoxication. The present study was designed to isolate, identify, and toxinotype *C. perfringens* from both healthy and cases of necrotic or ulcerative enteritis chickens. A total of 110 samples were collected from July 2019 to February 2021. Among the samples, 38 (34.5%, 95% CI: 26.39–43.83) were positive for *C. perfringens* and were obtained from broiler 21 (33.3%, 95% CI: 22.91–45.67), Sonali 9 (34.6%, 95% CI: 19.31–53.88), and layer 8 (38%, 95% CI: 20.68–59.20). *C. perfringens* was highly prevalent (35.7%, 95% CI: 25.48–47.44) in enteritis chickens compared with healthy ones. In multiplex PCR toxinotyping, 34 (89.4%) isolates were identified as *C. perfringens* type A by the presence of the alpha toxin gene (*cpa*). Moreover, in addition to the cpa gene, 3 (14.3%, 95% CI: 4.14–35.48) broiler and 1 (11.1%, 95% CI: 0.01–45.67) Sonali isolates harbored the enterotoxin gene (*cpe*) and were classified as type F. However, none of the isolates carried genes encoding beta (*cpb*), epsilon (*etx*), iota (*iap*), or beta-2 (*cpb2*) toxins. Multivariable logistic regression analysis identified the following variables such as; “previously used litter materials” (OR 21.77, 95% CI 2.22–212.66, *p* ≤ 0.008); intestinal lesions, “presence of ulceration” (OR 30.01, 95% CI 3.02–297.91, *p* ≤ 0.004); “ballooned with gas” (OR 24.74, 95% CI 4.34–140.86, *p* ≤ 0.001) and “use of probiotics” (OR 5.24, 95% CI 0.74–36.75, *p* ≤ 0.095) act as risk factors for *C. perfringens* colonization in chicken gut. This is the first study of molecular toxinotyping of *C. perfringens* from healthy and enteric-diseased chickens in Bangladesh, which might have a potential food-borne zoonotic impact on human health.

## 1. Introduction

*C. perfringens* is an anaerobic, spore forming enteric pathogen that causes both clinical and subclinical enteric disease in chickens. The most severe clinical form of enteric disease is necrotic enteritis, which is characterized by a ballooned, friable intestine with necrosis of the intestinal mucosa that is often covered by a tan-to-yellow pseudomembrane [1]. In addition, due to secondary bacterial infection and a roughened intestinal mucosal surface, it appears like a Turkish towel [1]. The clinical form of the disease is associated with a huge economic burden [2], and the subclinical form of the disease significantly reduces the growth performance of chickens by causing extensive damage to the gut epithelial layer [3]. The principal mechanism of disease manifestation by *C. perfringens* is associated with the release of six major extracellular toxins, which are described as alpha (*α*), beta (*β*), epsilon (*ε*), iota (*ι*), enterotoxin (*cpe*), and NetB. Based on the production of the abovementioned toxins, *C. perfringens* is classified into seven toxinogenic types, A to G [4, 5]. In the toxinogenic typing scheme, type A and all other types of *C. perfringens* produce alpha (a) toxin. In addition to alpha (*α*) toxin, type B produces beta (*β*) and epsilon (*ε*) toxins, type C produces beta (*β*) toxin, type D produces epsilon (*ε*) toxin, type E produces iota (*ι*) toxin, type F produces enterotoxin (*cpe*), and type G produces NetB toxin [4, 5]. Among all the *C. perfringens* types, type F is most frequently associated with food poisoning in humans and causes food-borne illness by producing an enterotoxin (*cpe*) [4–7]. Still, to date, it is positioned as the third most common food poisoning agent in the industrialized world [2].

Previously, the typing methodology for *C. perfringens* was based on a traditional toxin neutralization bioassay using mice or guineapigs [8]. Later, these typing schemes were replaced by enzyme-linked immunosorbent assays (ELISA) [9]. But, in the last few decades, *C. perfringens* typing schemes were accurately replaced by more convenient multiplex-polymerase chain reaction (M-PCR) assays targeting toxin-encoding genes [5, 10–13].

Although clinical necrotic enteritis cases are frequently found in poultry farms in Bangladesh, they are all diagnosed based only on clinical signs and postmortem findings. To date, this is the first study on the molecular identification of *C. perfringens,* which is circulating in chicken flocks in Bangladesh. As a result, the purpose of this study was to determine the phenotypic and toxinogenic typing of *C. perfringens* isolates from healthy and necrotic enteritis-infected chickens in Bangladesh.

## 2. Materials and Methods

### 2.1. Samples

**Figure 1:**
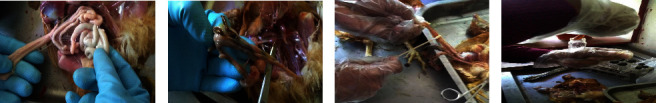
Postmortem examinations of chickens revealed the presence of intestinal lesions like (a) intestine balloons with gas, (b) ulceration, (c) collection of swab samples, and (d) intestinal contents for the isolation and identification of *C. perfringens*.

We collected intestinal swabs and intestinal contents from broiler, Sonali (a crossbreed of Fayoumi and Rhode Island Red), and layer chickens from July 2019 to February 2021. Samples were collected from chickens that were brought to the poultry practitioners for postmortem examinations at the Department of Pathology and Parasitology and the Department of Physiology, Biochemistry, and Pharmacology at Chattogram Veterinary and Animal Sciences University (CVASU). The samples only included chickens that showed enteric disease, including necrotic or ulcerative lesions during postmortem examination (Figures 1(a) and 1(b)). Additionally, specimens were collected from apparently healthy birds from live-broiler markets that were kept for selling. Intestinal swabs and intestinal contents (Figures 1(c) and 1(d)) were collected in 10 mL of cooked meat medium (Oxoid Limited, Hampshire, England) and immediately transported on ice to the Department of Microbiology and Veterinary Public Health laboratory for isolation and identification of *C. perfringens.*

### 2.2. Isolation and Identification of *C. perfringens*

For primary enrichment of *C. perfringens*, cooked meat medium containing samples was incubated anaerobically at 37°C for 48 hrs in an anaerobic jar (Oxoid Limited, Thermo Fisher Scientific Inc., UK) with an anaerobic GasPak (Oxoid Limited, Hampshire, England). The pre-enriched samples were then inoculated onto 5% bovine blood agar with added colistin sulfate (2 mg/litter) and incubated anaerobically for 24 hours. The characteristic colonies with double zones of beta hemolysis (inner zone: complete hemolysis; outer zone: partial hemolysis) were presumptively identified as *C. perfringens*. For further confirmation, the suspected bacterial colonies were subjected to Gram staining (Gram positive, large bacilli) and the catalase test (negative). Two to three subcultures were performed to obtain the pure culture, and finally, the pure colonies were subcultured on brain heart infusion broth (BHI) (Oxoid Limited, Thermo Fisher Scientific Inc., UK). All the isolates were preserved at −80°C for further analysis.

### 2.3. Extraction of DNA and Identification of *C. perfringens* Isolates by PCR

The preparation of template DNA was performed according to previously published literature [14]. Briefly, three to five pure bacterial colonies were suspended in 150 *μ*l of ultrapure water in a 1.5 ml microcentrifuge tube, boiled at 100°C for 10 min, and immediately cooled at −20°C for five minutes. Finally, the cell lysates were centrifuged at 12,000 *g* for 5 minutes, and 100 *μ*l of the supernatant containing template DNA was transferred into a microcentrifuge tube and stored at −20°C for further analysis. A species-specific primer (16S rRNA gene) was used for confirmation of *C. perfringens* [15]. A total of 25 *µ*l of the PCR reaction was prepared with 12.5 *µ*l of Taq PCR MasterMix (Qiagen, Merelbeke, Belgium), 1 *µ*l (20 picomole/*µ*l) of both forward and reverse primer, 4 *µ*l (5 ng/*µ*l) of template DNA, and 6.5 *µ*l of nuclease-free water. Amplification was performed in the thermal cycler (DLAB Scientific Inc., USA) with the PCR program consisting of initial denaturation at 95°C for 15 min, followed by 94°C for 30 sec, 53°C for 1.5 min, and 72°C for 1.5 min for 35 cycles, and final extension at 72°C for 10 min.

### 2.4. Toxinotyping by PCR

After confirmation of the *C. perfringens* species, all positive isolates were selected for toxinotyping using five different toxin genes *α* (*cpa*), *β* (*cpb*), *ε*(*etx*), *ί*(*iap*), and enterotoxin (*cpe*); their primer sequences are listed in Table 1. Multiplex PCR (m-PCR) was performed for specific amplification of the toxinotyping gene, which was described in a previous study [11]. For m-PCR, a total volume of 50 *µ*l reaction was prepared, which comprises 25 *µ*l of Taq PCR Master Mix, 1 *µ*l (20 pmol/*µ*l) of each primer for six toxin genes, 4 *µ*l of *C. perfringens* confirmed genomic DNA, and 9 *µ*l of nuclease-free water for every sample. A uniplex PCR was performed for the detection of *β*2 (*cpb-2*) and enterotoxin (*cpe*) gene, comprising 25 *µ*l of the reaction volume. Also, the similar PCR condition was followed to perform the uniplex PCR. Finally, all of the PCR products were separated on an ethidium bromide-stained 1.5% agarose gel (Oxoid Limited, Thermo Fisher Scientific Inc., UK) and visualized using UV lights in a gel documentation system (Alphalmager, Alpha Innotech, San Leandro, CA, USA). Previously confirmed *C. perfringens* isolates were used as positive controls, and nuclease-free water was used as a negative control.

### 2.5. Data Analysis

All targeted demographic as well as postmortem data was recorded into a Microsoft Excel 2010 spread sheet. The prevalence was calculated by considering the number of positive *C. perfringens* isolates as the numerator, divided by the number of chickens sampled as the denominator. Chi-square test was performed to find out the association between the binary result of *C. perfringens* and the farm and chicken factors. Firstly, univariable logistic regression analysis was performed to identify possible risk factors, and subsequently, any factor having a *p*-value of ≤0.20 was selected to build the further multivariable logistic regression model. Any variables with a *p*-value of 0.05 were considered significant and kept in the final model. All descriptive and analytical analyses were performed using STATA®13.0 software [16]. Finally, the representative heat map was constructed using Graphpad Prism (version 7.05).

## 3. Results

### 3.1. Samples

A total of 110 intestinal samples were collected from broiler (63), Sonali (26), and layer (21) chickens; 40 samples from healthy broiler and 70 from enteric-diseased chicken with various intestinal lesions.

### 3.2. Prevalence of *C. perfringens*

**Figure 2:**
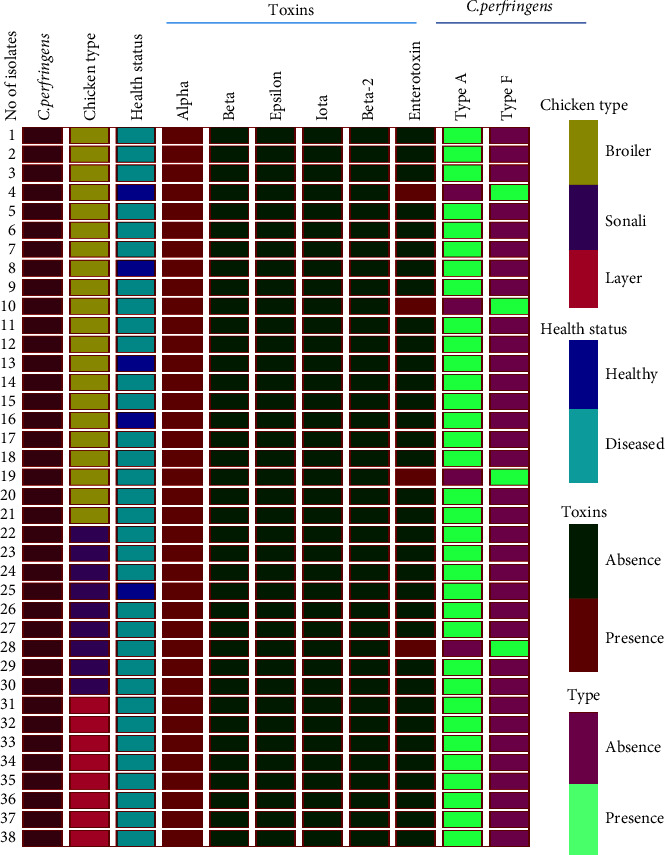
Heat map illustrates the chicken types and their health status as well as the distribution of different *C. perfringens* types and their toxins.

A total of 38 (34.5%, 95% CI: 26.39 to 43.83) PCR-confirmed *C. perfringens* isolates were recovered from 110 chickens (Table 2). Of them, 21 (33.3%, 95% CI: 22.91 to 45.67) isolates were obtained from broiler, 9 (34.6%, 95% CI: 19.31 to 53.88) from Sonali, and 8 (38%, 95% CI: 20.68 to 59.20) from layer chickens (Figure 2). Among the *C. perfringens* isolates, 13 (32.5%, 95% CI: 20.01 to 48.06) were recovered from healthy broiler chickens, and 25 (35.7%, 95% CI: 25.48 to 47.44) were from diseased chickens (Table 2).

### 3.3. Toxin Typing and Distribution of *C. perfringens*

All *C. perfringens* isolates (38) encode the alpha toxin gene (*cpa*) (Table 2), and 34 (89.4%) are classified as type A (Figure 2). The type A *C. perfringens* was distributed among the broiler, Sonali, and layer chickens, which were 18 (47.3%), 8 (21.0%), and 8 (21.0%), respectively. In addition to the alpha toxin gene (*cpa*), 4 (10.5%) isolates also harbored the enterotoxin gene (*cpe*), which is classified as type F (Figure 2). Moreover, these enterotoxin-producing isolates were obtained from Sonali (1) and broiler (3) chickens (Figure 2). Of the three enterotoxin-positive isolates from broiler chickens, one isolate was recovered from healthy chickens, and the remaining two isolates were found in diseased chickens. None of the isolates encoded beta, iota, epsilon, or beta 2-toxin genes (Figure 2).

### 3.4. Risk Factors Associated with the Harboring of *C. perfringens* in Enteric Diseased Chicken

The univariable analysis identified five potential risk factors (*p* ≤ 0.20) associated with the harboring of *C. perfringens* in enteric diseased chickens (Table 3). In the subsequent multivariable analysis, three variables were identified as significant risk factors associated with the presence of *C. perfringens.* The significantly associated variables were: “previously used litter materials” (OR 21.77, 95% CI 2.22–212.66, *p* ≤ 0.008), intestinal lesions “ulceration” (OR 30.01, 95% CI 3.02–297.91, *p* ≤ 0.004) and “ballooned with gas” (OR 24.74, 95% CI 4.34–140.86, *p* ≤ 0.000), and “use of probiotics” (OR 5.24, 95% CI 0.74–36.75, *p* ≤ 0.095) (Table 4).

## 4. Discussion

*C. perfringens* are spore-forming bacilli that commonly inhabit soil, poultry litter, and are also harbored as gut pathogens in chickens and other animals [17, 18]. The different toxinotypes cause a wide variety of significant systemic and enteric diseases in different species of animals, including chickens. Another significant aspect is that it is involved in food-borne intoxication (food-borne zoonosis), which evolved from consumption of different raw and canned foods, particularly chicken meat and meat products [19].

In the present study, the overall prevalence of *C. perfringens* in chicken is 34.5%, but in some countries, particularly Jordan and Egypt, it is higher than 40% [20, 21]. Besides, the prevalence of *C. perfringens* in broiler chicken was 33.3% in our study, which is slightly higher than the recent findings of Praveen Kumar et al. [22] and Zhang et al. [23]. They described the prevalence as 21.97% and 23.1% in broiler chicken in India and China, respectively. This variation may be due to geographical conditions as well as the earlier contaminated soil and unchanged bedding materials of the poultry farm. As *C. perfringens* is a spore-forming pathogen that can survive in soil for an extended period of time, chickens are frequently infected. On the other hand, chickens that harbor *C. perfringens* continuously shed it in the poultry litter and environment, and it acts as a potential source of infection for chickens. Cross-infection through droppings, litter materials, poultry waste products, feed, and water is a major route of infection for chickens and other animals [23, 24]. In addition, the prevalence of *C. perfringens* may increase significantly when chickens have a concurrent clinical or subclinical coccidial infection in the gut [25].

Toxinotyping of *C. perfringens* by multiplex PCR assay displayed the presence of the alpha toxin gene in all isolates and the absence of other major lethal toxin genes except the enterotoxin (*cpe*) gene, which was expressed by type F isolates. The current findings demonstrate that 34 (89.4%) of the PCR-confirmed *C. perfringens* isolates are type A. This study revealed that *C. perfringens* type A is the most dominant toxin type circulating both in healthy and diseased chickens. These findings are strongly supported by several previous studies [2, 20, 23, 26]. However, none of the *C. perfringens* isolates carried the *cpb*, *etx*, or *iap* toxin genes, which indicate the absence of *C. perfringens* toxinotypes B, C, D, and E in this study. Similar findings were also previously reported [26–28], showing that *C. perfringens* obtained from chickens encoded only the alpha toxin gene. In addition to the four major toxinotyping toxins, *β*2 toxin (*cpb2*) was also absent in all chicken isolates, which were considered minor or nonessential virulence factors for disease manifestation in chicken [29].

Although *C. perfringens* is a significant food-borne zoonotic pathogen, very limited microbiological and epidemiological studies have been reported in Bangladesh. In our study, 4 (10.5%) of the type F isolates harbored the enterotoxigenic gene (*cpe*), which is a potential virulence determinant of *C. perfringens* for food poisoning in humans [4, 5, 19, 30]. The low incidence of enterotoxin in chicken may be due to sample source variation as well as the genetic absence of an encoded gene. However, in addition to food-borne intoxication, the enterotoxin producing *C. perfringens* strains also frequently cause neonatal diarrhea [31]. Furthermore, the enterotoxin gene (*cpe*) encoded by *C. perfringens* type F isolates clearly poses the risk of human food-borne intoxication as well as food-borne zoonosis [4, 5, 32]. Finally, it is recognized that any *C. perfringens* isolates with alpha toxin and enterotoxin have a significant impact on the production of necrotic enteritis in chickens and also cause fatal food-borne enteric disease in humans. However, in the current study, we classified *C. perfringens* from A to F, with the exception of type G, which harbored a net B toxin.

We identified previously used litter materials as a potential risk factor for the colonization of *C. perfringens* in chickens. Besides, the presence of associated lesions, such as ulceration and ballooned gas in the chicken gut, is also asserted as a colonization factor. Long-term and repeated use of the same litter materials in poultry houses may harbor the spores of *C. perfringens* [23, 33] and act as an initial trigger of exposure. The presence of intestinal lesions may involve the initial intestinal damage caused by coccidial pathogens [34] and subsequent colonization of *C. perfringens*. Finally, *C. perfringens* releases various toxins and enzymes responsible for gut lesions in chicken. Moreover, further details on the sequencing of *C. perfringens* isolates helped to find out the mechanisms of colonization and toxin production, as well as the control of necrotic enteritis in chickens and food-borne zoonosis in humans.

## 5. Conclusions

This study describes the prevalence and toxinotyping of *C. perfringens* isolated from chicken in Bangladesh. We found that 33.3% of broiler, 34.6% Sonali, and 38.0% of layer chickens tested positive for *C. perfringens.* It is interesting that, among the enteric-diseased and healthy chickens, the prevalence of *C. perfringens* was higher in the enteric-diseased birds (35.7%). In the present investigation, alpha toxin-producing *C. perfringens* type A was the predominant genotype, and relatively considerable numbers of isolates were type F (10.5%) that carried the enterotoxin gene (*cpe*). Previously used litter materials were identified as risk factors that were associated with a higher prevalence of *C. perfringens* in chickens.

## Figures and Tables

**Table 1:** Oligonucleotide primer sequences and amplified PCR product sizes were used for the detection of *C. perfringens* and its toxin genes.

Name of bacteria and toxins	Target gene	Primers name	Primer sequences (5′-3′)	PCR product size (bp)	Ref
*C. perfringens*	16*S RNA*	ClPer-1	TAACCTGCCTCATAGAGT	481	[15]
		ClPer-2	TTTCACATCCCACTTAATC		

Alpha (*α*)	*cpa*	CPAlphaF	GCTAATGTTACTGCCGTTGA	324	[11]
		CPAlphaR	CCTCTGATACATCGTGTAAG		
Beta (*β*)	*cpb*	CPBetaF3	GCGAATATGCTGAATCATCTA	195	
		CPBetaR3	GCAGGAACATTAGTATATCTTC		
Epsilon (*ε*)	*etx*	CPEpsilonF	TGGGAACTTCGATACAAGCA	376	
		CPEpsilonR2	AACTGCACTATAATTTCCTTTTCC		
Iota (*ι*)	*iap*	CPIotaF2	AATGGTCCTTTAAATAATCC	272	
		CpIotaR	TTAGCAAATGCACTCATATT		
Beta-2 (*β*2)	*cpb2*	CPBeta2totalF2	AAATATGATCCTAACCAACAA	548	
		CPBeta2totalR	CCAAATACTCTAATYGATGC		
Enterotoxin	*cpe*	CPEnteroF	TTCAGTTGGATTTACTTCTG	485	
		CPEnteroR	TGTCCAGTAGCTGTAATTT		

**Table 2:** Distribution of *C. perfringens* isolates and its toxins from different chickens.

Chickens	No. of samples		PCR positive isolates						
	Live bird market (healthy)	Clinical case (diseased)	Cp^a^(%)	*α*	*β*	*ε*	*ι*	*β*2	Et^b^(%)
Broiler	40	23	21 (33.3)	21	0	0	0	0	3 (14.3)
Sonali	0	26	9 (34.6)	9	0	0	0	0	1 (11.1)
Layer	0	21	8 (38.0)	8	0	0	0	0	0
Total	110		38 (34.5)	38	0	0	0	0	4 (10.5)

^a^ *Clostridium perfringens*, ^b^enterotoxin.

**Table 3:** Univariable logistic regression analysis of risk factors for the presence of *C. perfringens* in diseased chicken.

Variables	Covariable	No. of chickens	No. of chickens positive for *C. perfringens*(%)	95% CI^a^	OR (95% CI)	*p*-value^b^
Farm location	Hilly area	6	1 (16.67)	0.42–64.12	Reference	0.309
	Plain land	64	24 (37.50)	25.70–50.49	3 (0.33–27.23)	

Birds type	Layer	21	8 (38.10)	18.10–61.56	1.16 (0.35–3.84)	0.964
	Broiler	23	8 (34.78)	16.37–57.26	1 (0.30–3.27)	
	Sonali	26	9 (34.62)	17.21–55.66	Reference	

Flock size^c^	Small	9	3 (33.33)	7.48–70.07	1.3 (0.23–7.31)	0.674
	Medium	18	5 (27.78)	9.69–53.48	Reference	
	Large	43	17 (39.53)	24.97–55.59	1.7 (0.13–1.07)	

Chicken age^d^	Chick	3	0 (0.00)	—	1.71 (0.02–2.23)	0.619
	Pullet	4	2 (50.00)	6.75–93.24	4 (0.21–75.65)	
	Finisher	5	1 (20.00)	0.50–71.64	Reference	
	Hen	14	6 (42.86)	17.66–71.13	3 (0.26–34.19)	
	Starter	20	6 (30.00)	11.89–54.27	1 (−)	
	Grower	24	10 (41.67)	22.10–63.35	2.85 (0.27–29.56)	

Feed types	Homemade	7	3 (42.86)	9.89–81.59	0.73 (0.14–3.65)	0.907
	Mash	15	5 (33.33)	11.82–61.61	Reference	
	Pellet	48	17 (35.42)	22.16–50.54	0.66 (0.10–4.20)	

Litter materials	No use	11	3 (27.27)	6.02–60.97	3.37 (0.46–24.28)	0.006^b^
	New	20	2 (10.00)	1.23–61.69	Reference	
	Previously used	39	20 (35.71)	34.78–67.58	9.47 (1.93–46.46)	

Materials type	Straw	5	3 (60.00)	14.66–94.72	4 (0.43–37.10)	0.606
	Cage	11	3 (27.27)	6.02–60.97	Reference	
	Rice husk	15	6 (40.00)	16.33–67.02	1.33 (0.30–5.88)	
	Saw dust	39	13 (33.33)	19.08–50.21	1.77 (0.33–9.55)	

Floor type	Muddy	5	2 (40.00)	5.27–85.33	1.70 (0.24–11.95)	0.475
	Semi pucca	32	9 (28.13)	13.74–46.74	Reference	
	Concrete	33	14 (42.42)	25.47–60.78	1.88 (0.66–5.29)	

Water source	Pond	1	0 (0.00)	—	Omitted	0.730
	Tube well	29	10 (34.48)	17.93–54.33	Reference	
	Tank water	40	15 (37.50)	22.72–54.19	1.14 (0.42–3.09)	

Vaccination	Yes	61	22 (36.07)	24.16–49.37	1.12 (0.25–4.96)	0.873
	No	9	3 (33.33)	7.48–70.07	Reference	

Deworming	Yes	25	9 (36.00)	17.97–57.47	1.01 (0.36–2.82)	0.970
	No	45	16 (35.56)	21.86–51.21	Reference	

Use of antibiotics	Yes	54	23 (42.59)	29.23–56.79	5.19 (1.07–25.13)	0.027
	No	16	2 (12.50)	1.55–38.34	Reference	

Use of probiotics	Yes	13	7 (53.85)	25.13–80.77	2.52 (0.74–8.60)	0.131
	No	57	18 (31.58)	19.90–45.24	Reference	

Use of feed enzymes	Yes	31	12 (38.71)	21.84–57.81	1.26 (0.47–3.37)	0.641
	No	39	13 (33.33)	19.08–50.21	Reference	

Use of feed supplements	Yes	63	24 (38.10)	26.14–51.20	3.69 (0.41–32.57)	0.212
	No	7	1 (14.29)	0.36–57.87	Reference	

Intestinal lesions	Ballooned with gas	5	2 (40.00)	5.27–85.33	6.22 (0.72–53.37)	0.001^b^
	Ulceration	13	5 (38.46)	13.85–68.42	5.83 (1.13–29.85)	
	Necrosis	21	15 (71.43)	47.82–88.71	23.33 (5.09–106.81)	
	Hemorrhage	31	3 (9.68)	2.04–25.75	Reference	

^a^-Confidence interval, ^b^-significance, which is ≤0.05, ^c^-flock size which are small (≤500), medium (501–1000) and large (≥1000), ^d^-chicken age: in case of broiler and Sonali, [starter (1–21 days), grower (22–42) and finisher (≥42 days)]; in case of layer, [chick (1–28 days), pullet (29–105 days), and hen (≥105 days)].

**Table 4:** Multivariable logistic regression analysis of risk factors for the presence of *C. perfringens* in diseased chicken.

Outcome variable	Explanatory variable	Description	OR^*∗*^(95%CI)	*p*-value
*Clostridium perfringens*	Litter materials	No use	5	0.218
		New	1	Reference
		Previously used	21.77 (2.22–212.66)	0.008
	Intestinal lesions	Hemorrhage	1	Reference
		Ulceration	30.01 (3.02–297.91)	0.004
		Necrosis	3.17 (0.22–44.04)	0.388
		Ballooned with gas	24.74 (4.34–140.86)	0.001
	Use of probiotics	No	1	Reference
		Yes	5.24 (0.74–36.75)	0.095

^*∗*^Odds ratio.

## Data Availability

The data used to support the findings of this study are available from the corresponding author upon request.
